# The cytochrome P450 CYP6DE1 catalyzes the conversion of α-pinene into the mountain pine beetle aggregation pheromone *trans*-verbenol

**DOI:** 10.1038/s41598-018-38047-8

**Published:** 2019-02-06

**Authors:** Christine C. Chiu, Christopher I. Keeling, Joerg Bohlmann

**Affiliations:** 1 Michael Smith Laboratories, University of British Columbia, 2185 East Mall, Vancouver, B.C. V6T 1Z4 Canada; 2 Department of Botany, University of British Columbia, 6270 University Blvd, Vancouver, B.C. V6T 1Z4 Canada; 3 Present Address: Laurentian Forestry Centre, Canadian Forest Service, Natural Resources Canada, 1055 rue du P.E.P.S., P.O. Box 10380, Stn. Sainte-Foy, Québec, QC G1V 4C7 Canada

## Abstract

The recent outbreak of the mountain pine beetle (*Dendroctonus ponderosae;* MPB) has affected over 20 M hectares of pine forests in western North America. During the colonization of host trees, female MPB release the aggregation pheromone (−)-*trans*-verbenol. (−)-*trans*-Verbenol is thought to be produced from the pine defense compound (−)-α-pinene by cytochrome P450 (P450) dependent hydroxylation. MPB may also use P450s for the detoxification of other monoterpenes of the pine defense system. Here we describe the functional characterization of MPB CYP6DE1. CYP6DE1, but not the closely related CYP6DE2, used the bicyclic monoterpenes (−)-α-pinene, (+)-α-pinene, (−)-β-pinene, (+)-β-pinene and (+)-3-carene as substrates. CYP6DE1 was not active with other monoterpenes or diterpene resin acids that were tested as substrates. *trans*-Verbenol is the major product of CYP6DE1 activity with (−)-α-pinene or (+)-α-pinene as substrates. When tested with blends of different ratios of (−)-α-pinene and (+)-α-pinene, CYP6DE1 produced *trans*-verbenol with an enantiomeric profile that was similar to that produced by female MPB exposed to the α-pinene enantiomers.

## Introduction

The mountain pine beetle (*Dendroctonus ponderosae*; MPB) is a pest of several pine (*Pinus*) species in western North America^1^. While endemic MPB populations are mostly confined to weakened host trees, during epidemic outbreaks MPB successfully colonize healthy mature trees^2,3^. Pines and other conifers are generally well defended against most herbivores by their production, accumulation and secretion of oleoresin terpenes^4–6^. However, conifer-feeding bark beetles and their associated microbiomes have evolved mechanisms to detoxify host terpenes^7–9^. Bark beetles may also co-opt metabolized host terpenes as signal molecules for their intraspecific communications^10,11^.

**Figure 1:**
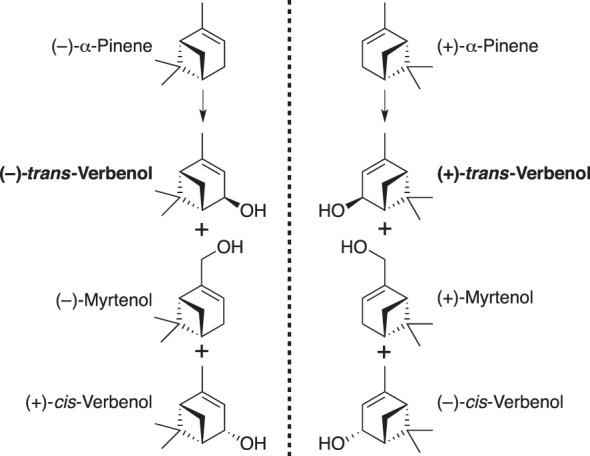
The two enantiomers of α-pinene and their hydroxlated products *trans*-verbenol, myrtenol and *cis*-verbenol. (−)-α-Pinene is the precursor of (−)-*trans*-verbenol, (−)-myrtenol and (+)-*cis*-verbenol. (+)-α-Pinene is the precursor of (+)-*trans*-verbenol, (+)-myrtenol and (−)-*cis*-verbenol.

MPB can tolerate high concentrations of oleoresin monoterpenes^12^, which they experience throughout the life cycle from egg to adult and during different phases of activity from host colonization to dispersal. MPB also use volatile host monoterpenes as cues during the dispersal flight to locate new hosts^13^. Another chemo-ecological feature of MPB is their release of the aggregation pheromone *trans*-verbenol during host colonization^14,15^. (−)-*trans*-Verbenol [(1*S*, 2*R*, 5*S*)-4,6,6-trimethylbicyclo[3.1.1]hept-3-en-2-ol] is a hydroxylated derivative of (−)-α-pinene, and is the active enantiomer, while (+)-*trans*-verbenol [(1*R*, 2*S*, 5*R*)-4,6,6-trimethylbicyclo[3.1.1]hept-3-en-2-ol], which is inactive as a pheromone in MPB, is derived from (+)-α-pinene (Fig. 1). Both (−)-α-pinene and (+)-α-pinene are common monoterpenes of the oleoresin and volatile emissions of pines^16,17^. The enantiomeric ratio of α-pinene in the oleoresin of the MPB host lodgepole pine (*Pinus contarta*) is approximately 70% (−)- and 30% (+)-α-pinene^17,18^. In jack pine (*P. banksiana*) the enantiomeric ratio is approximately 20% (−)-and 80% (+)-α-pinene^17,18^.

It was previously thought that only female MPB produce, and immediately release, *trans*-verbenol by oxidation of α-pinene upon landing on a new host tree^11,19^. However, we recently showed that both male and female MPB accumulate verbenol as fatty acid esters during their juvenile life stages, suggesting that formation of verbenol and verbenyl esters occurs in both sexes possibly as part of monoterpene detoxification in the feeding larvae^20^. While both sexes accumulate verbenyl esters as larvae, only females retain these metabolites when they emerge as adults from the brood tree and may use them as a source for sex-specific pheromone release^20^.

The enzymes and biochemical processes involved in MPB monoterpene detoxification and terpenoid pheromone formation may be, at least in part, identical^21,22^. The oxygenation of a lipophilic compound such as a monoterpene will result in a more polar metabolite which may be excreted, become the substrate for further conjugation, or may be otherwise metabolized^23^. It is conceivable that ancestors of MPB first evolved genes and enzymes for monoterpene detoxification as an essential process to survive conifer host defenses. Such a detoxification resulted in the formation and release of monoterpenoid derivatives by beetles that may have been co-opted as pheromones. A prominent gene family in the MPB genome that may serve functions in terpene detoxification and pheromone biosynthesis as well as in olfaction of monoterpenes are the cytochromes P450 (P450s)^24^. Of the 86 P450s identified in the MPB genome^24^, only five have been reported as functionally characterized for their substrates and products. Specifically, CYP345E2, an antennae-specific P450 epoxidizes or hydroxylates (+)-3-carene, (−)-camphene and both enantiomers of α-pinene, β-pinene and limonene^25^. Using either enantiomer of α-pinene as substrate, CYP345E2 produces α-pinene oxide^25^. CYP6DE3 also uses (+)-α-pinene, 3-carene and (+)-limonene as substrates^21^. It produces 3-oxatricyclo [4.1.1.0(2, 4)] octane and (+)-*trans*-verbenol from (+)-α-pinene^21^. CYP6CR1 epoxidizes (*Z*)-6-nonen-2-one to 6,7-epoxynonan-2-one, a precursor to the male MPB pheromone *exo*-brevicomin^26^. CYP4G55/56 convert both long chain and short chain alcohols to aldehydes and decarbonylate the aldehydes to hydrocarbon and carbon dioxide as part of cuticular hydrocarbon and exo-brevicomin production^27^.

In a recent transcriptome screen of the MPB P450 gene family we identified P450s that are expressed in different body parts where monoterpene oxidation may occur, specifically in antennae for olfaction, as well as in the alimentary canal and fat body for detoxification and pheromone formation^22^. Of the seven different MPB P450s identified in this screen, transcripts of *CYP6DE1* were highly abundant in antennae and fat body. The closest related gene family member *CYP6DE2* (83% translated amino acid identity) was most highly expressed in antennae and midgut tissues. Transcripts of *CYP6DE3* (72% and 65% translated amino acid identity with CYP6DE1 and CYP6DE2, respectively) were more abundant in unfed males compared to fed males, unfed and fed females^21^.

Here we describe the heterologous expression of CYP6DE1 and CYP6DE2 proteins and their reconstitution with MPB cytochrome P450 reductase (CPR) for functional characterization. CYP6DE1 was active with (−)-α-pinene, (+)-α-pinene, (−)-β-pinene, (+)-β-pinene and (+)-3-carene as substrates, and it produced *trans*-verbenol from blends of (−)-α-pinene and (+)-α-pinene with an enantiomeric product profile that closely resembled that of female MPB exposed to the same enantiomeric α-pinene blend.

## Results

### CYP6DE1 converts some, but not all, host monoterpenes and is not active with diterpene resin acids

**Figure 2:**
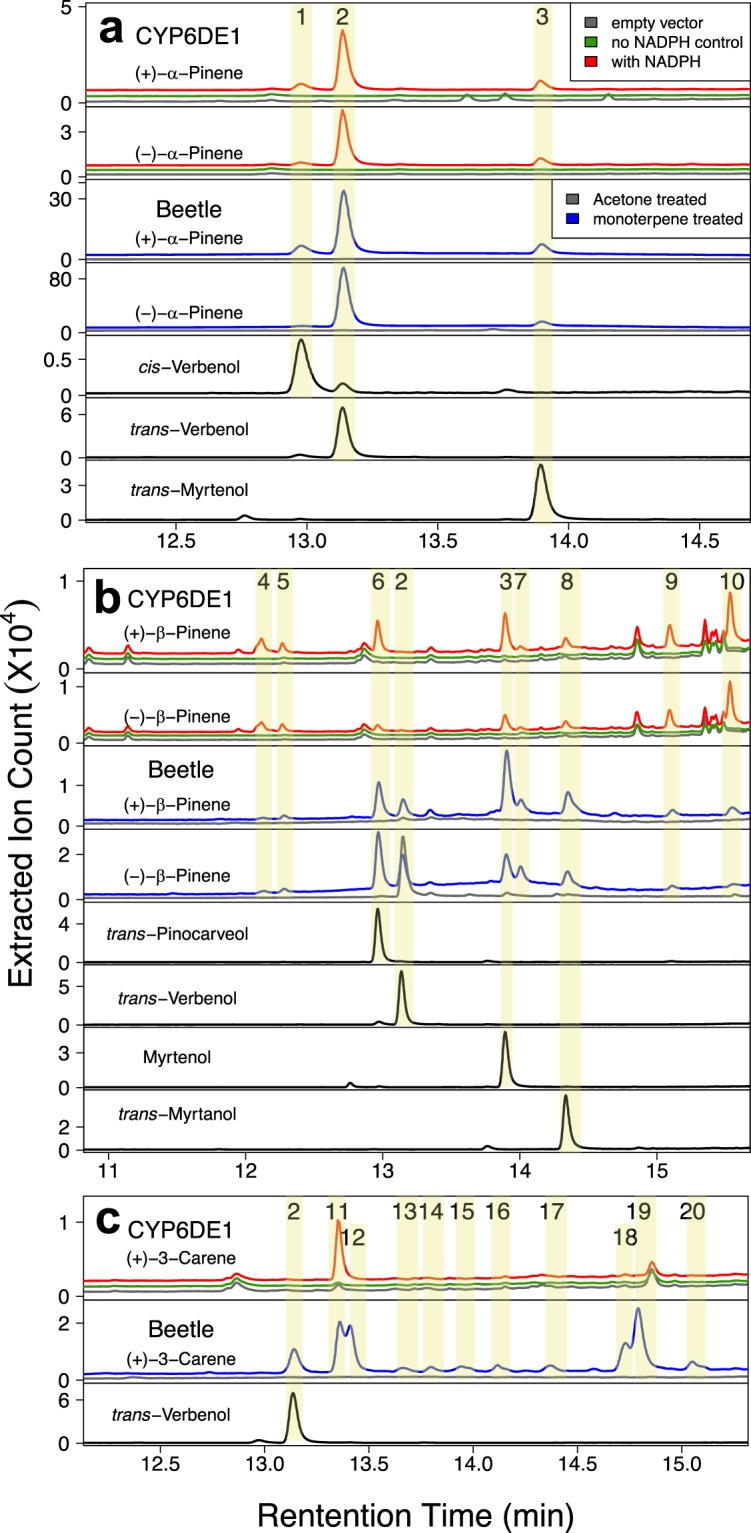
GC-MS traces of products formed by CYP6DE1 in *in vitro* enzyme assays with different monoterpenes, and GC-MS traces of products extracted from female MPB exposed to different monoterpenes. Enzyme assays and beetle treatments with (**a**) (+)-α-pinene or (−)-α-pinene; (**b**) with (+)-β-pinene or (−)-β-pinene; and (**c**) with (+)−3-carene. Treatment of beetles with acetone was performed as a control. Enzyme assays included controls without CYP6DE1 (empty vector) or without NADPH. Peak 1 is *cis*-verbenol; peak 2 is *trans*-verbenol, peak 3 is myrtenol; peak 6 is *trans*-pinocarveol; peak 8 is *trans*-myrtanol. Peaks 4, 5, 7, 9–21 represent unidentified products. Representative GC-MS traces are shown with the total of the extracted ions 91, 94, 108, 109, 119, 121, 137, 152. Retention indices and mass spectra are shown in Supporting Table S2 and Supporting Figs S3–S7.

MPB CYP6DE1 and CYP6DE2 were produced in *Spodoptera frugiperda* cells, isolated as microsomal membrane-bound proteins (Supporting Fig. S1), and identified as functional P450s based on CO-spectra (Supporting Fig. S2). Both P450s were reconstituted with MPB CPR and tested in *in vitro* enzyme assays with ten different monoterpenes and five different diterpene resin acids (DRAs). The substrates that we tested represent typical monoterpenes and DRAs found in the phloem of the MPB host lodgepole pine^16,28,29^. While we did not detect activity of CYP6DE2 with any of the substrates tested (Supplemental Table 1), CYP6DE1 oxidized both enantiomers of the bicyclic monoterpenes α-pinene and β-pinene as well as (+)-3-carene (Fig. 2; Supporting Table S1). Assay products with CYP6DE1 were identified by comparison of retention times and mass-spectra with those of authentic standards (Supporting Figs S3–S7; Supporting Table S2). In assays with (+)-α-pinene or (−)-α-pinene, CYP6DE1 produced *cis*-verbenol (peak 1), *trans*-verbenol (peak 2) and myrtenol (peak 3) (Fig. 2a; Supporting Fig. S3). Products from CYP6DE1 assays with (+)-β-pinene or (−)-β-pinene were myrtenol (peak 3), *trans*-pinocarveol (peak 6), and *trans*-myrtanol (peak 8), along with five other unidentified peaks (peaks 4, 5, 7, 9 and 10) (Fig. 2b; Supporting Figs S4–S6). The product from CYP6DE1 assays with (+)-3-carene was a single unidentified peak (peak 11) (Fig. 2c; Supporting Fig. S7). These oxygenated monoterpenoid products were not detected in assays with the empty vector control, or with CYP6DE1 assays that did not contain NADPH (Fig. 2; Supporting Fig. S3–S7). CYP6DE1 was not active with the other five monoterpenes tested and was also not active with any of the diterpenes tested (Supporting Table S1).

### CYP6DE1 kinetics with α-pinene

Kinetic parameters *K*_m_, *k*_cat_ and *V*_max_ of CYP6DE1 were in the same order of magnitude with (+)-α-pinene and (−)-α-pinene as substrates (Table 1; Supporting Figs S8 and S9). The enzyme appeared to be slightly more efficient *in vitro* with (−)-α-pinene with a catalytic efficiency (*k*_cat_/*K*_m_) of 2324 ± 919 s^−1^ M^−1^, compared to *k*_cat_/*K*_m_ of 576 ± 262 s^−1^ M^−1^ with (+)-α-pinene.

**Table 1:** Kinetic properties of CYP6DE1.

	*K*_m_ (µM)	*V*_max_ (µM s^−1^)	*k*_cat_ (s^−1^)	*k*_cat_/*K*_m_ (s^−1^ M^−1^)
(+)-α-Pinene	292 ± 1	0.007 ± 0.003	0.17 ± 0.08	576 ± 262
(−)-α-Pinene	160 ± 1	0.016 ± 0.006	0.37 ± 0.15	2324 ± 919

### Product profiles of CYP6DE1 match products of female MPB exposed to monoterpenes

**Figure 3:**
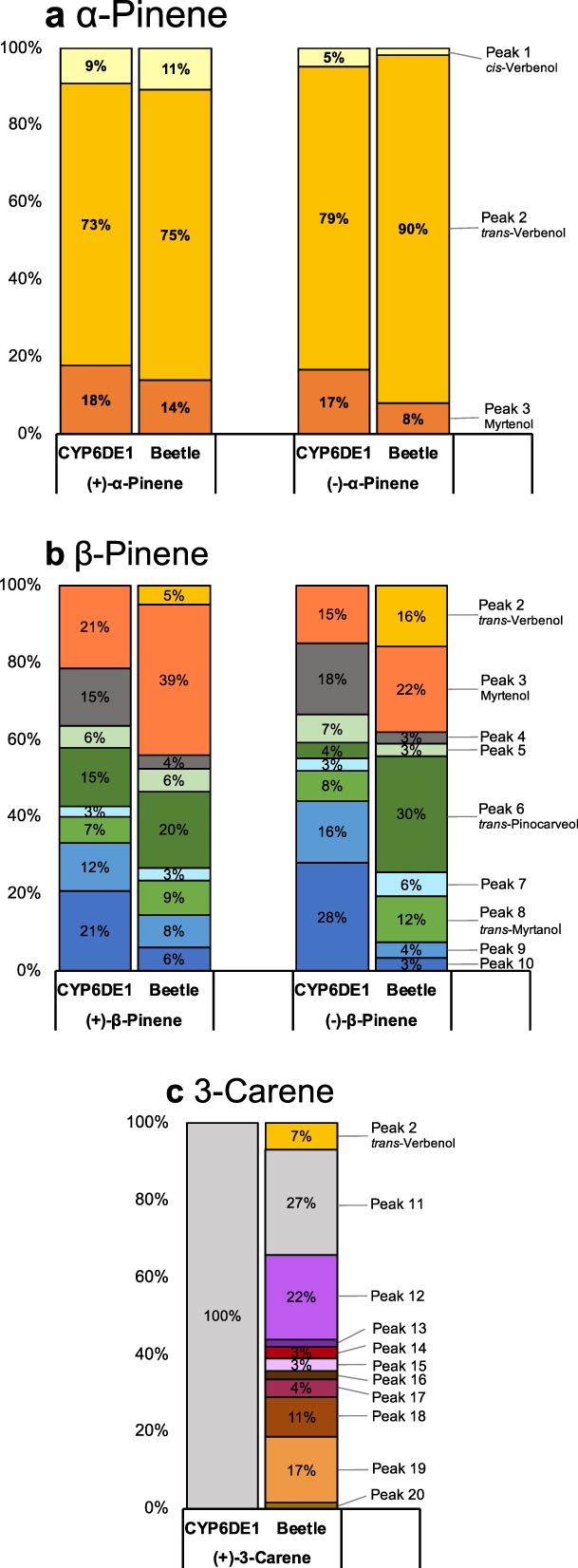
Relative quantitative composition of products formed by CYP6DE1 in *in vitro* enzyme assays with different monoterpenes, and GC-MS traces of products extracted from female MPB exposed to different monoterpenes. (**a**) The percentage profile was calculated from total nanograms based upon response factors of authentic standards. (**b**,**c**) The percentage profile was calculated by peak area of the extracted ion chromatogram. Retention indices and mass spectra are shown in Supporting Table S2 and Supporting Figs S3–S7. CYP6DE1 assays were performed with N = 6 replicates; MPB treatment assay were performed with N = 4 replicates.

Since the activity of CYP6DE1 with (+)-α-pinene, (−)-α-pinene, (+)-β-pinene, (−)-β-pinene, and (+)-3-carene resulted in multiple products for each substrate, we measured the relative amounts of each product (Fig. 3). We then compared the product composition formed by CYP6DE1 *in vitro* with products extracted from female MPB that were exposed to the same five different monoterpenes. Both the beetles and CYP6DE1 showed regioselectivity and diastereoselectivity for the different substrates and their enantiomers.

Overall, the proportion of products formed by CYP6DE1 with (+)-α-pinene or (−)-α-pinene closely resembled the monoterpenols found in MPB exposed to the same monoterpenes (Figs 2a and 3a). Extracts of MPB exposed to (+)-α-pinene contained *cis*-verbenol (peak 1), *trans*-verbenol (peak 2) and myrtenol (peak 3) (Fig. 2a). These metabolites were not identified in extracts from females exposed to acetone serving as a negative control (Fig. 2a). The relative composition of the product profile of (+)-α-pinene-treated MPB was 11% *cis*-verbenol, 75% *trans*-verbenol and 14% myrtenol (Fig. 3a). For comparison, the product profile of CYP6DE1 assays with (+)-α-pinene consisted of 9% *cis*-verbenol, 73% *trans*-verbenol and 18% myrtenol (Fig. 3a).

Beetles that were exposed to (−)-α-pinene also contained *cis*-verbenol (peak 1), *trans*-verbenol (peak 2) and myrtenol (peak 3) (Fig. 2a). The product profile of (−)-α-pinene-treated MPB consisted of 2% *cis*-verbenol, 90% *trans*-verbenol and 8% myrtenol (peak 3) (Fig. 3a). The product profile of CYP6DE1 assays with (−)-α-pinene contained 5% *cis*-verbenol, 79% *trans*-verbenol (peak 2) and 17% myrtenol (Fig. 3a).

MPB exposed to (+)-β-pinene contained *trans*-verbenol (peak 2), myrtenol (peak 3), *trans*-pinocarveol (peak 6), *trans*-myrtanol (peak 8), and five unidentified peaks (peaks 4, 5, 7, 9 and 10) (Fig. 2b). Of these peaks, only peak 2 (*trans*-verbenol) was recorded in controls in which females had been exposed to acetone, presumably due to prior formation of this metabolite^20^ (Fig. 2b). The terpenol profile of (+)-β-pinene-treated MPB consisted of 5% *trans*-verbenol, 39% myrtenol, 4% peak 4, 6% peak 5, 20% *trans*-pinocarveol, 3% peak 7, 9% *trans*-myrtanol, 8% peak 9, and 6% peak 10. (Fig. 3b). For comparison, the product profile of CYP6DE1 assays with (+)-β-pinene consisted of 21% myrtenol, 15% peak 4, 6% peak 5, 15% *trans*-pinocarveol, 3% peak 7, 7% *trans*-myrtanol, 12% peak 9, and 21% peak 10 (Fig. 3b).

MPB exposed to (−)-β-pinene contained *trans*-verbenol (peak 2), myrtenol (peak 3), *trans*-pinocarveol (peak 6), *trans*-myrtanol (peak 8), and five unidentified peaks (peaks 4, 5, 7, 9 and 10) (Fig. 2b). The product profile of (−)-β-pinene-treated MPB consisted of 16% *trans*-verbenol, 22% myrtenol, 3% peak 4, 3% peak 5, 30% *trans*-pinocarveol, 6% peak 7, 12% *trans*-myrtanol, 4% peak 9, and 3% peak 10 (Fig. 3b). For comparison, the product profile of CYP6DE1 assays with (−)-β-pinene consisted of 15% myrtenol, 18% peak 4, 7% peak 5, 4% *trans*-pinocarveol, 3% peak 7, 8% *trans*-myrtanol, 16% peak 9, and 28% peak 10 (Fig. 3b).

MPB exposed to (+)-3-carene contained *trans*-verbenol (peak 2) and nine other unidentified peaks (peaks 11–20) (Fig. 2c). Peak 2 (*trans*-verbenol) was not recorded in controls with acetone treated females (Fig. 2c). The product profile of (+)-3-carene-treated beetle extracts consisted of 7% *trans*-verbenol, 27% peak 11, 22% peak 12, 2% peak 13, 3% peak 14, 3% peak 15, 2% peak 16, 4% peak 17, 11% peak 18, 17% peak 19, and 2% peak 20 (Fig. 3c). The product profile of CYP6DE1 assays with (+)-3-carene consisted of 100% peak 11 (Fig. 3c).

### *In vitro* activity of CYP6DE1 and female MPB show minor enantiomeric preferences in their formation of *trans*-verbenol

**Figure 4:**
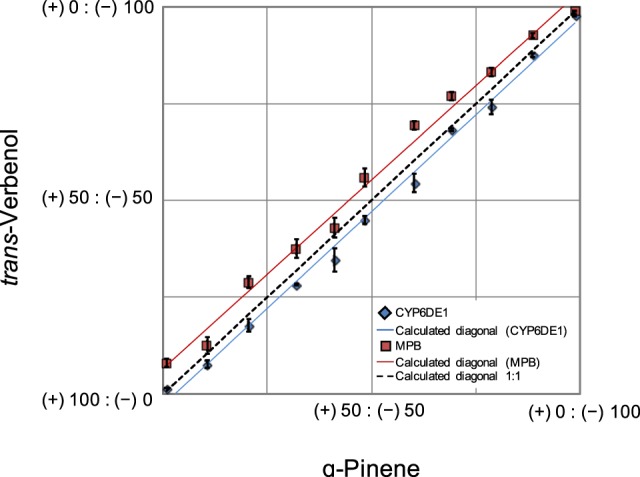
The proportion of (−)-*trans*-verbenol and (+)-*trans*-verbenol produced by CYP6DE1 or female MPB in assays or treatments, respectively, with different ratios of (−)-α-pinene and (+)-α-pinene. A series of enantiomeric ratios of (−)-α-pinene and (+)-α-pinene were used as either a substrate for CYP6DE1 enzyme assays or in treatment assays with female MPB. The enantiomeric ratios of the corresponding *trans*-verbenol products were measured. CYP6DE1 assays were performed with N = 3 replicates; MPB treatment assay were performed with N = 3 replicates.

The utilization of both enantiomers of α-pinene by CYP6DE1 was of particular interest for two reasons: first, the product profiles of CYP6DE1 with these substrates closely matched the products observed in female MPB exposed to the same compounds (Fig. 3a); and second, only the (−) enantiomer of α-pinene can yield the pheromone-active (−) enantiomer of *trans*-verbenol (Fig. 1). In a host tree such as lodgepole pine, MPB are exposed to both enantiomers of α-pinene and their ratios may vary by pine species and genotype^16,30^. We therefore tested the *in vitro* activity of CYP6DE1 with different enantiomeric ratios of α-pinene, and we compared the enantiomeric composition of the *trans*-verbenol products with those detected in female MPB exposed to the same enantiomeric ratios of α-pinene (Fig. 4). At all enantiomeric ratios of α-pinene tested, the enantiomeric ratios of *trans*-verbenol produced were similar in *in vivo* assays with female MPB and in *in vitro* assays with CYP6DE1. In female MPB exposed to different enantiomeric ratios of α-pinene the enantiomeric ratio of *trans*-verbenol showed a minor shift towards a slightly higher proportion of the (−) enantiomer in the product compared to the substrate. Differences in the enantiomeric ratio of (−)-*trans*-verbenol produced compared to the enantiomeric ratio of (−)-α-pinene used to treat beetles was tested using a *t*-test (*p* < 0.05) and found to be significant (*t* = 8.0726, *df* = 32, *p*-value < 0.001). Using the same enantiomeric ratios of α-pinene as substrate in *in vitro* assays with CYP6DE1 we found a minor enantiomeric preference towards formation of the (+) enantiomer. Differences in the proportion of (−)-*trans*-verbenol produced compared to the proportion (−)-α-pinene given as a substrate were tested using a *t*-test (*p* < 0.05) and found to be significant (*t* = −5.3198, *df* = 30, *p*-value < 0.001).

## Discussion

We showed that CYP6DE1 has a biochemical function in the oxidation of the bicyclic monoterpenes (−)-α-pinene, (+)-α-pinene, (−)-β-pinene, (+)-β-pinene, and (+)-3-carene. To our knowledge, these results are the first report of a bark beetle P450 to produce (−)-*trans*-verbenol, a monoterpenol that serves as a pheromone in the aggregation biology of the MPB. The MPB P450 CYP6DE3 was previously reported to convert (+)-α-pinene into (+)-*trans*-verbenol^21^, but assays with (−)-α-pinene were not reported. It is therefore not known whether CYP6DE1 and CYP6DE3 have overlapping or distinct biochemical functions in their activity with the two enantiomers of α-pinene. Unlike CYP6DE3^21^, CYP6DE1 did not show 3-oxatricyclo [4.1.1.0(2, 4)] octane as a product.

In contrast to CYP6DE1 and CYP6DE3, no enzyme activity is known for CYP6DE2, which may require different assay conditions to be active *in vitro*. The use of several, but not all, monoterpenes as substrates suggest that CYP6DE1 may serve a general role in monoterpene detoxification and monoterpene removal, while it may also contribute to pheromone biosynthesis. Testing the possible biological roles of CYP6DE1 further *in vivo* will require gene editing or RNAi knock down experiments. We have successfully used RNAi with a few other MPB genes^31^, but we have not been able to apply RNAi with conclusive results for CYP6DE1.

The monoterpenol product profiles of CYP6DE1 with (+)-α-pinene or (−)-α-pinene were similar to those found in female MPB exposed to these monoterpenes, both in terms of qualitative and quantitative composition. CYP6DE1 produced mostly *trans*-verbenol with minor amounts of *cis*-verbenol and myrtenol, similar to the monoterpenol composition released by female beetles as an aggregation pheromone when colonizing a new host tree^32^. By comparison, the products profiles of CYP6DE1 with (+)-β-pinene, (−)-β-pinene or (+)-3-carene were substantially different in their quantitative or qualitative composition from the monoterpenols extracted from beetles treated with the same compounds. These results suggest that additional P450s are active in MPB and may contribute to the conversion of (+)-β-pinene, (−)-β-pinene and (+)-3-carene, while CYP6DE1 appears to be a major contributing enzyme in the conversion of (+)-α-pinene or (−)-α-pinene in MPB.

In MPB exposed to β-pinene, (+)-3-carene and in some of the MPB exposed to acetone (control), we also observed *trans*-verbenol at levels that were over 30-fold lower than the amount of *trans*-verbenol released by beetles exposed to α-pinene. Previous reports also showed that MPB exposed to acetone may sometimes release small amounts of trans-verbenol^20,33,34^. Those releases of trans-verbenol, which appear to be variable, may be due to the prior formation and sequestration of this compound in the form of its fatty acid esters during the early life stages prior to the experiments^20^. We did not detect *trans*-verbenol in assays of CYP6DE1 with β-pinene or (+)-3-carene.

(+)-β-Pinene, (−)-β-pinene, and (+)-3-carene are of mid-range toxicity to MPB compared to other common monoterpenes of pine oleoresin^12,35^. Previous work showed that MPB feeding on jack pine with a monoterpene profile dominated by (+)-3-carene produced less *trans*-verbenol than MPB feeding on jack pine with a monoterpene profile dominated by (+)-α-pinene^32^. Future work may explore the effect of (−)-α-pinene, (+)-β-pinene, (−)-β-pinene, and (+)-3-carene as potentially competing substrates of CYP6DE1 on trans-verbenol formation in MPB.

The aggregation pheromone produced by female MPB consists of 87–97% (−)-*trans*-verbenol compared to (+)-*trans*-verbenol^18,20,33^. We tested if adult female MPB in *in vivo* assays, or CYP6DE1 in *in vitro* assays, would be selective for producing (−)-*trans*-verbenol over (+)-*trans*-verbenol. Adult female MPB showed only a minor preference for producing the (−) enantiomer, while CYP6DE1 had a similarly minor preference for the (+) enantiomer. The CYP6DE1 *in vitro* kinetic parameters were similar for the two enantiomers of α-pinene, and CYP6DE1 appeared to be only slightly more catalytically efficient with (−)-α-pinene. Thus, the predominantly (−) enantiomeric ratio of *trans-verbenol* released by adult females may originate in the processes of formation, accumulation, and hydrolysis of the verbenyl esters^20^, and would not be explained by the minor stereoselectivity of adult female MPB or CYPDE6 with α-pinene.

**Figure 5:**
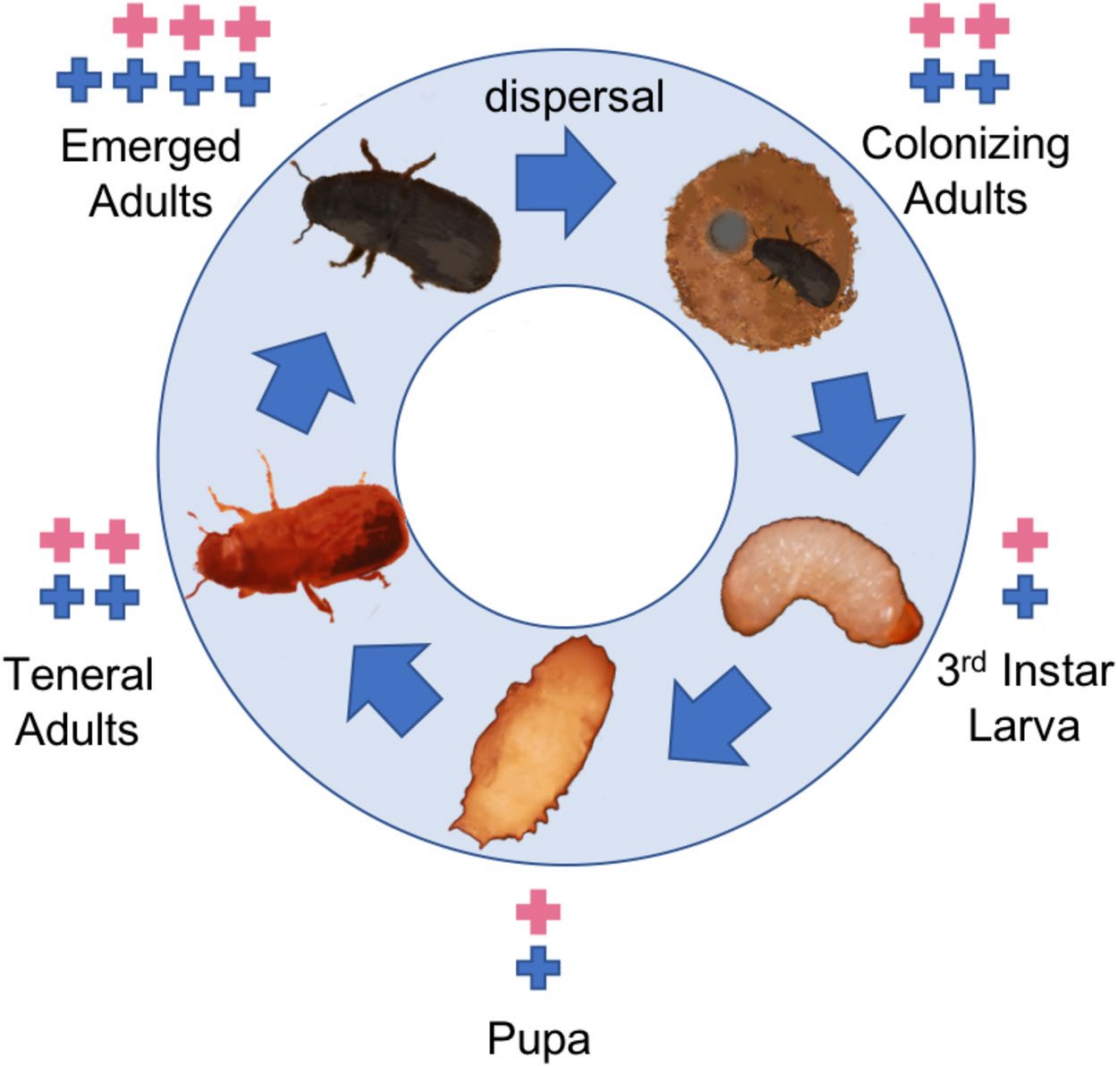
Schematic of transcript abundance of *CYP6DE1* over the lifecycle of the MPB. *CYP6DE1* is expressed in both sexes (blue + symbols for males; pink + symbols for females) throughout the life cycle. Relative transcript abundance is illustrated with one (lowest) to four (highest) + symbols. The schematic is based on data reported in Chiu *et al*.^20^ and Chiu *et al*.^22^.

*CYP6DE1* transcripts are expressed in female and male MPB at different stages of the life cycle, including larvae, pupae, teneral adults, freshly emerged adults and host colonizing adults (Fig. 5)^22^. In both sexes of emerged and colonizing MPB, *CYP6DE1* transcripts are expressed most abundantly in antennae and fat body, and at relatively lower levels in the midgut^22^. Female and male MPB accumulate verbenyl esters during the juvenile life stages from larvae to teneral adults, and in emerging adult females, verbenyl esters are most abundant in the abdomen and specifically in the fat body surrounding the alimentary canal^20^. The combined knowledge on transcript profiles and biochemical functions of CYP6DE1, together with the recent discovery of accumulation of verbenyl esters in the early life stages of female and male MPB^20^, suggest that CYP6DE1 may be involved in the formation of verbenyl esters via hydroxylation of α-pinene.

## Methods

### Insects

MPB infested lodgepole pine (*P. contorta*) trees were felled near Whistler, BC, Canada (50°12′33.3″N 122°53′05.2″W) in October 2015 and (50°12′46.6″N 122°53′20.8″W) in September 2016. Stems were cut into bolts, which were placed in screened cages and stored indoors at the University of British Columbia at room temperature. Emerged beetles were collected every three to four days and sexed based on abdominal tergite shape^36^.

### Chemicals

Chemicals obtained from Sigma-Aldrich (Mississauga, ON, Canada) were: *N*,*O*-*bis*(trimethylsilyl)trifluoroacetamide (BSTFA, cat. No. 15209), methyl *tert*-butyl ether (MTBE, cat. No. 650560), pentane (cat. No. 34956), (+)-α-pinene (cat. No. P45680), and (−)-α-pinene (cat. No. 274399), (+)-β-pinene (cat. No. 80607), (−)-β-pinene (cat. No. 112089), (+)-3-carene (cat. No. 441619), (−)-limonene (cat. No. 218367), (+)-limonene (cat. No. 62122), myrcene (cat. No. M100005), terpinolene (cat. No. 86485), (−)-*trans*-myrtanol (cat. No. W343900), and (−)-*trans*-pinocarveol (cat. No. 80613). Chemicals obtained from Helix Biotech (Richmond, BC, Canada) were: abietic acid (cat. No. R002), dehydroabietic acid (cat. No. R001), neoabietic acid (cat. No. R003), levopimaric acid (cat. No. R005), pimaric acid (cat. No. R011). Chemicals obtained from PheroTech (Delta, BC, Canada) were: *trans*-verbenol (approx. 20(+):80(−) enantiomeric purity) (lot. No. W06-00141) and *cis*-verbenol (approx. 20(+):80(−) enantiomeric purity) (lot. No. CV001129). The (−)-*trans*-myrtanol (cat. No. 5134S) was from Extrasynthese (Genay, France). The (−)-β-phellandrene was obtained by purification from lodgepole pine turpentine by Synergy Semiochemicals (Burnaby, Canada).

### Heterologous expression of CYP6DE1, CYP6DE2, and CPR

The full-length open reading frames of *CYP6DE1* (JQ855668, DPO0814_E19) and *CYP6DE2* (JQ855669, DPO047_M21)^37^ were cloned into the pFastBac vector (Invitrogen) and transformed into MAX Efficiency DH10Bac Competent cells (Invitrogen, cat. # 10361-012) to generate recombinant bacmids. Empty pFastBac vector was used to generate a recombinant bacmid for negative controls. Bacmids were used to transfect *Spodoptera frugiperda Sf9* cells (Invitrogen, cat. # 1265-017) for production of baculovirus to a titer of 2–5 × 10^7^ IFU × mL^−1^. The resulting baculovirus was used to infect 250 mL of Sf9 cell culture (cell density 7.5 × 10^6^ cells × mL^−1^) at a ratio of baculovirus to Sf9 cells of 1:1. The pelleted seed culture was incubated with the baculovirus culture for 1 h at 27 °C and then resuspended in 250 mL of Sf-900 II serum-free media (Invitrogen, cat # 10902-088) with 10% fetal bovine serum. Hemin HCl (Sigma cat #51280) was added to a concentration of 2 μg mL^−1^ 24 h after the infection. Cells were harvested 72 h after infection. Cells were pelleted and washed three times with 50 mM potassium phosphate buffer (KPB), pH7.4. Cells were resuspended in P450 buffer (50 mM of KPB pH 7.4, 20% glycerol, 1 mM EDTA and 0.1 mM DTT), disrupted by sonification, and centrifuged for 1 h at 100,000 × *g* to collect the microsomes. Microsomes were suspended in 5 mL of P450 buffer and aliquots were frozen at −80 °C until use. To test for the presence of the P450 proteins, 5 μL of denatured microsomes were analyzed on a 12% SDS-PAGE gel, and P450 activity was checked by carbon monoxide (CO)-difference spectrum analysis^38,39^. MPB CPR (JQ855645) was expressed in *E. coli* as previously described^25^.

### Enzyme assays

*In vitro* assays with CYP6DE1 or CYP6DE2 were performed individually with ten different monoterpenes [(+)-α-pinene, (−)-α-pinene, (+)-β-pinene, (−)-β-pinene, (+)-limonene, (−)-limonene, (+)-3-carene, myrcene, β-phellandrene, and terpinolene] and five different diterpene resin acids (DRAs; abietic acid, dehydroabietic acid, neoabietic acid, levopimaric acid, and pimaric acid). Assays with microsomes from empty vector expression, as well as assays with CYP6DE1 or CYP6DE2 microsomes without NADPH, were used as negative controls. Microsomes from P450 or empty vector expression were combined with CPR microsomes and kept on ice for 1 h before being used in assays. Assays were prepared as follows: 25 µL of P450 microsome (0.5–2.0 µM) and 2 µL of CPR microsome (1 U/mL) were added to 2-mL amber glass vials (Agilent, cat# 5182-00716), and KPB (pH 7.4) and NADPH were added for a final concentration of 50 mM KPB and 1 mM NADPH. To start the assays, 3 µL of individual monoterpenes (10 mM in pentane) or DRAs (1 mM in MTBE) was added and the vial was immediately capped. The total assay volume was 300 µL. Assays were incubated for 1 h at 30 °C and then extracted three times with pentane (for assays with monoterpenes) or MTBE (for assays with DRAs). Extracts were concentrated under a N_2_ stream to 300 µL. Extracts from assays with DRAs were derivatized by adding 5 µl of BSTFA to 50 µL of assay extract and letting the sample incubate overnight. Assay products were analyzed by gas chromatography coupled mass spectroscopy (GC-MS). Assays to determine enzyme kinetics of CYP6DE1 were performed in triplicate with (+)-α-pinene and (−)-α-pinene at concentrations of 25 µM, 50 µM, 100 µM, 200 µM, 400 µM, 600 µM, 800 µM, and 1200 µM. Assays were incubated for 1 h, then immediately frozen in liquid N_2_, and kept frozen until extraction with pentane. Under these conditions, product formation maintained linearity for at least 100 min. Kinetic parameters were determined by nonlinear regression with the Michaelis-Menten model using ANEMONA^40^.

### Treatment of MPB with α-pinene, β-pinene and 3-carene

Female MPB used in this experiment were from lodgepole pine bolts collected in October 2015. Emergent beetles were exposed to vapours of (+)-α-pinene, (−)-α-pinene, (+)-β-pinene, (−)-β-pinene, or (+)-3-carene corresponding to 0.1 µL volume of monoterpene per mL of airspace as previously described in Chiu *et al*.^12^. A 1.5 cm × 1.5 cm Whatman filter paper was placed into a 20 mL scintillation vial (VWR) and 2 µL of the individual monoterpene was added to the filter paper. For controls, 1 µL of acetone was used instead of monoterpene. Beetles were placed into the vials with one beetle per vial, and vials were capped. After 24 h living beetles were collected, frozen with liquid N_2_ and kept at 80 °C until extraction. Each beetle was extracted individually and monoterpene and monoterpenols analyzed by GC-MS as previously described^20^. Treatment experiments were performed with four replicates for each monoterpene, each replicate consisting of a single female beetle.

### Tests for the stereoselectivity of MPB and CYP6DE1 with α-pinene

Female MPB used in this experiment were from lodgepole pine bolts collected in September 2016. Treatments of beetles with monoterpenes were performed as described above, except instead of individual monoterpenes, mixtures of (+)-α-pinene and (−)-α-pinene were used at different ratios with 2 µL of blend placed on a 1.5 cm × 1.5 cm Whatman filter paper in a 20 mL glass vial. The enantiomeric ratios of (+)-α-pinene: (−)-α-pinene were 10:0, 9:1, 8:2, 7:3, 6:4, 5:5, 4:6, 3:7, 2:8, 1:9, 0:10. Three replicates were performed for each ratio, each replicate consisting of a single female beetle. For comparison, enzyme assays with CYP6DE1 were performed as described above with the same enantiomeric mixtures, with 3 µL of 1 mM substrate in pentane added to the 300 µL total assay volume. Enzyme assays were performed with three replicates. Data from tests of the stereoselectivity of MPB and CYP6DE1 with α-pinene by were analyzed for differences in the proportion of (−)-*trans*-verbenol produced compared to the proportion (−)-α-pinene given as a substrate tested using a *t*-test (p < 0.05).

### GC-MS analysis

GC-MS analyses were performed on an Agilent 7890A system GC, Agilent GC Sampler 80, and a 7000A GC/MS triple quad M5975C inert XL MSD with triple axis detector at 70 eV. Monoterpenes and monoterpenols from the enzyme assays and treatment of MPB were analyzed by injecting 1 µL of sample onto a DB-WAX column (J&W, polyethylene glycol, 30 m, 250 μm i.d., 0.25 μm film thickness). Oven temperature for analysis of products from enzyme assays with monoterpenes was 40 °C for 2 min, 8 °C min^−1^ to 100 °C, 20 °C min^−1^ to 230 °C and then held for 5 min. Oven temperature for analysis of beetle extracts was 40 °C for 2 min, 8 °C min^−1^ to 100 °C, 20 °C min^−1^ to 250 °C and then held for 10 min. Products from enzyme assays with DRAs were analyzed by injecting 1 µL of derivatized sample onto a HP-5 column (J&W, 5% phenyl methyl siloxane, 27.4 m length, 250 μm i.d., 0.25 μm film thickness). Oven temperature for analysis of products from enzyme assays with DRAs was 40 °C for 1 min, 20 °C min^−1^ to 300 °C and then held for 8 min. Stereochemistry of α-pinene and *trans*-verbenol from the enzyme assays and MPB treatment with mixtures of (+)-α-pinene and (−)-α-pinene were analyzed by injecting 1 µL of sample onto a CyclodexB column (10.5% β-cyclodextrin, 25.7 m length, 250 μm i.d., 0.25-μm film thickness). Oven temperature for analysis of products from enzyme assays and MPB extracts was 40 °C for 2 min, increase at 10 °C min^−1^ to 100 °C, 20 °C min^−1^ to 230 °C, hold for 7 min with pulsed splitless injector held at 250 °C.

## Supplementary information

Supplementary Information

## Data Availability

All data generated or analysed during this study are included in this published article and its Supplementary Information files. Any additional data generated during and/or analysed during the current study are available from the corresponding author on reasonable request.
