# Supplementary material for: The cytochrome P450 CYP6DE1 catalyzes the conversion of α-pinene into the mountain pine beetle aggregation pheromone trans-verbenol
Source: Sci Rep. 2019 Feb 6;9:1477. doi: 10.1038/s41598-018-38047-8 (PMC6365528; doi:10.1038/s41598-018-38047-8)
Supplement: Supplementary file 1 — Supplementary Information [file 41598_2018_38047_MOESM1_ESM.pdf]

The cytochrome P450 CYP6DE1 catalyzes the conversion of α-pinene into the mountain pine beetle aggregation pheromone *trans*-verbenol

Christine C. Chiu[1,2], Christopher I. Keeling[1,3], Joerg Bohlmann[1,2*]

[1]Michael Smith Laboratories, University of British Columbia, 2185 East Mall, Vancouver, B.C., Canada, V6T 1Z4

[2]Department of Botany, University of British Columbia, 6270 University Blvd, Vancouver, B.C., V6T 1Z4

[3]Present address: Laurentian Forestry Centre, Canadian Forest Service, Natural Resources Canada, 1055 rue du P.E.P.S., P.O. Box 10380, Stn. Sainte-Foy, Québec, QC, Canada, G1V 4C7

[*]corresponding author: E-mail: bohlmann@msl.ubc.ca

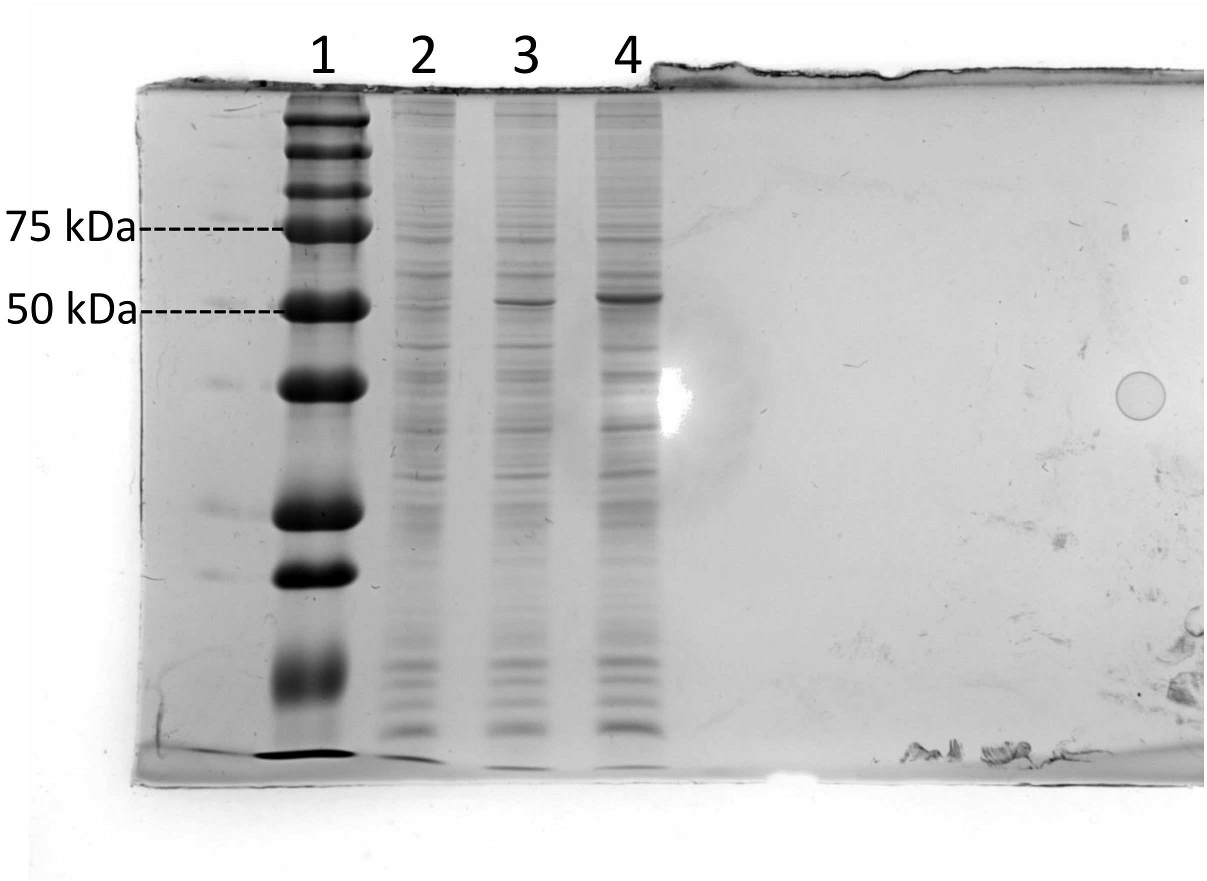

Figure S1. Denatured CYP6DE1, CYP6DE2 and empty vector control microsomes on a 12% SDS-PAGE gel. P450s were heterologously expressed in Sf9 cells. Lane 1: Precision Plus Protein ladder (Bio-rad). Lane **2**: empty vector microsomes. Lane **3**: CYP6DE1 microsomes, protein band is visible between 75 kDa and 50 kDa. Lane **4**: CYP6DE2 microsomes, protein band is visible between 75 kDa and 50 kDa. Original uncropped image is shown.

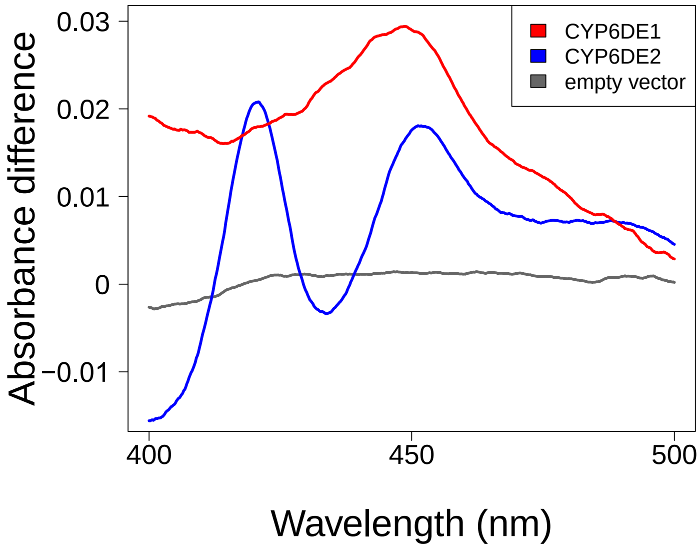

Figure S2. CO Spectra of CYP6DE1, CYP6DE2 and empty vector control microsomes. P450 microsomes that were harvested from Sf9 cells were tested for P450 activity according to Omura and Sato (1964). Absorbance difference is the difference in absorbance between reduced P450 microsomes and CO-bound P450 microsomes. CYP6DE1 and CYP6DE2 both produce a peak at 450 nm, indicating active P450 enzymes. CYP6DE2 also produces a peak at 420 nm, indicating inactive P450 enzymes. The empty vector control does not produce a peak at 420 or 450 nm, indicating the lack of abundant P450 enzymes in these microsomes.

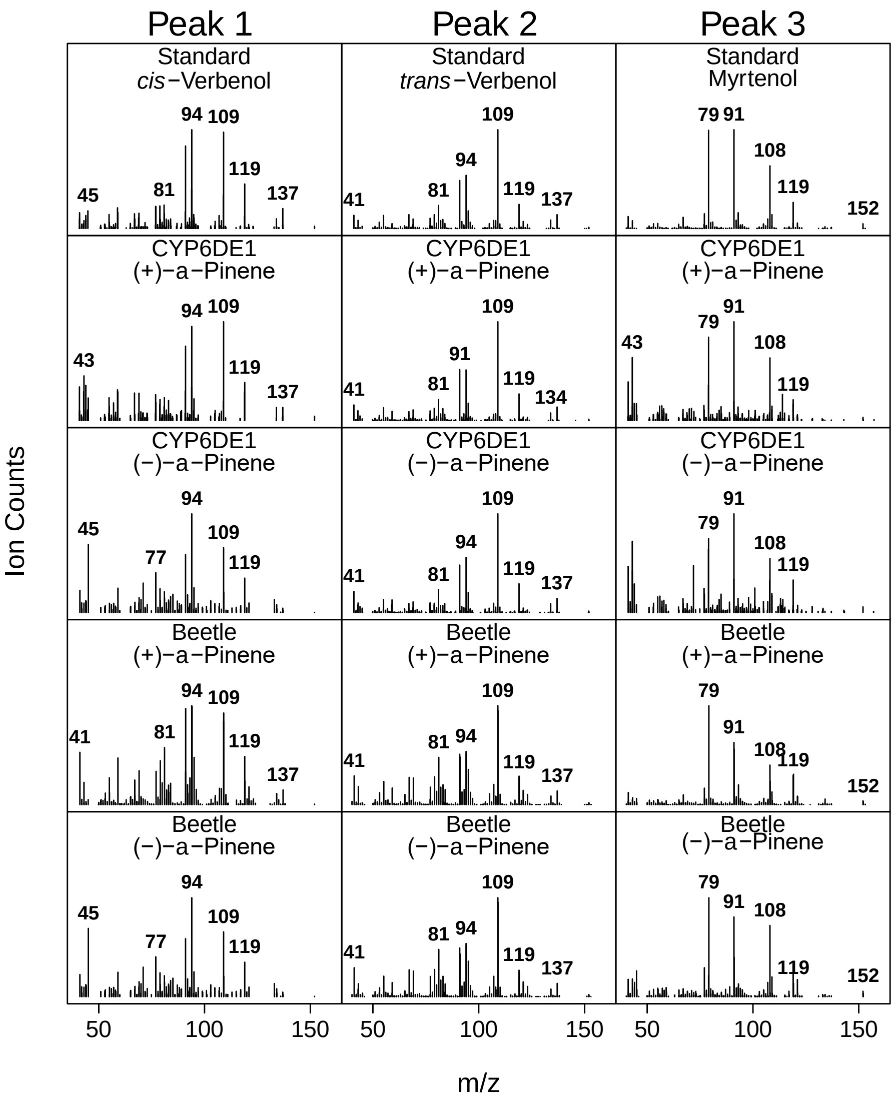

Figure S3 Mass spectra of peaks 1-3 from the gas chromatograms of extracts of CYP6DE1 or female beetles treated with (+) and (-)-α-pinene along with the *cis* and *trans*-verbenol and myrtenol standards. Gas chromatograms with peak numbers can be found in Fig. 2A.

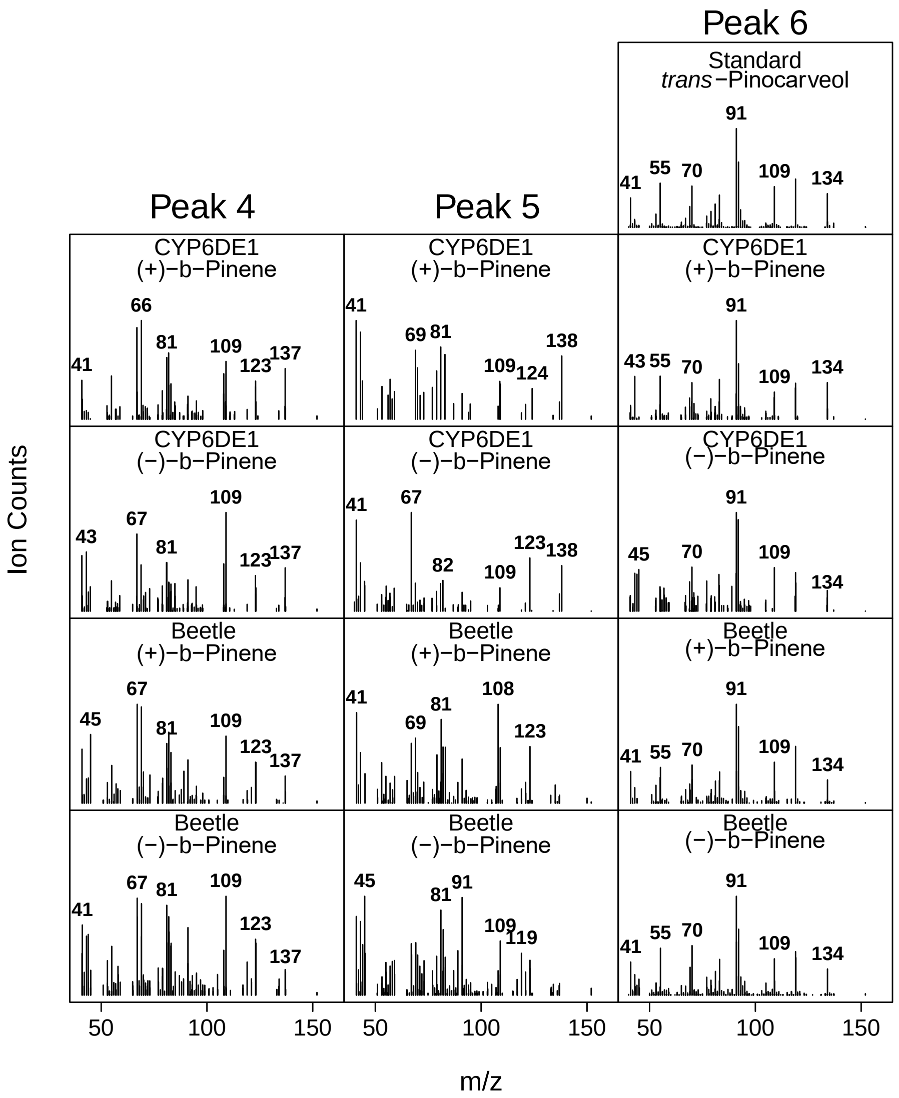

Figure S4 Mass spectra of peaks 4-6 from the gas chromatograms of extracts of CYP6DE1 or female beetles treated with (+) and (-)-β-pinene along with the *trans*-pinocarveol standard. Gas chromatograms with peak numbers can be found in Fig. 2B.

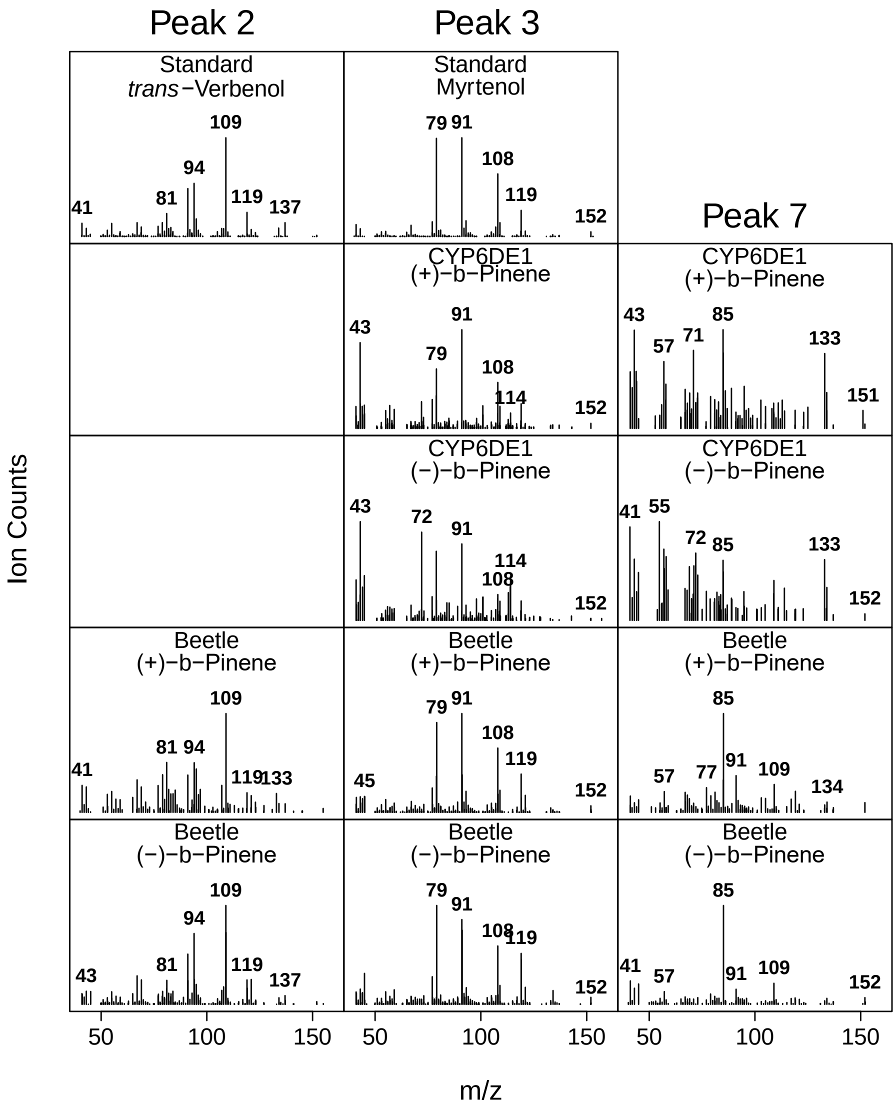

Figure S5 Mass spectra of peaks 2,3,7 from the gas chromatograms of extracts of CYP6DE1 or female beetles treated with (+) and (-)-β-pinene along with the *trans*-verbenol and myrtenol standards. Gas chromatograms with peak numbers can be found in Fig. 2B.

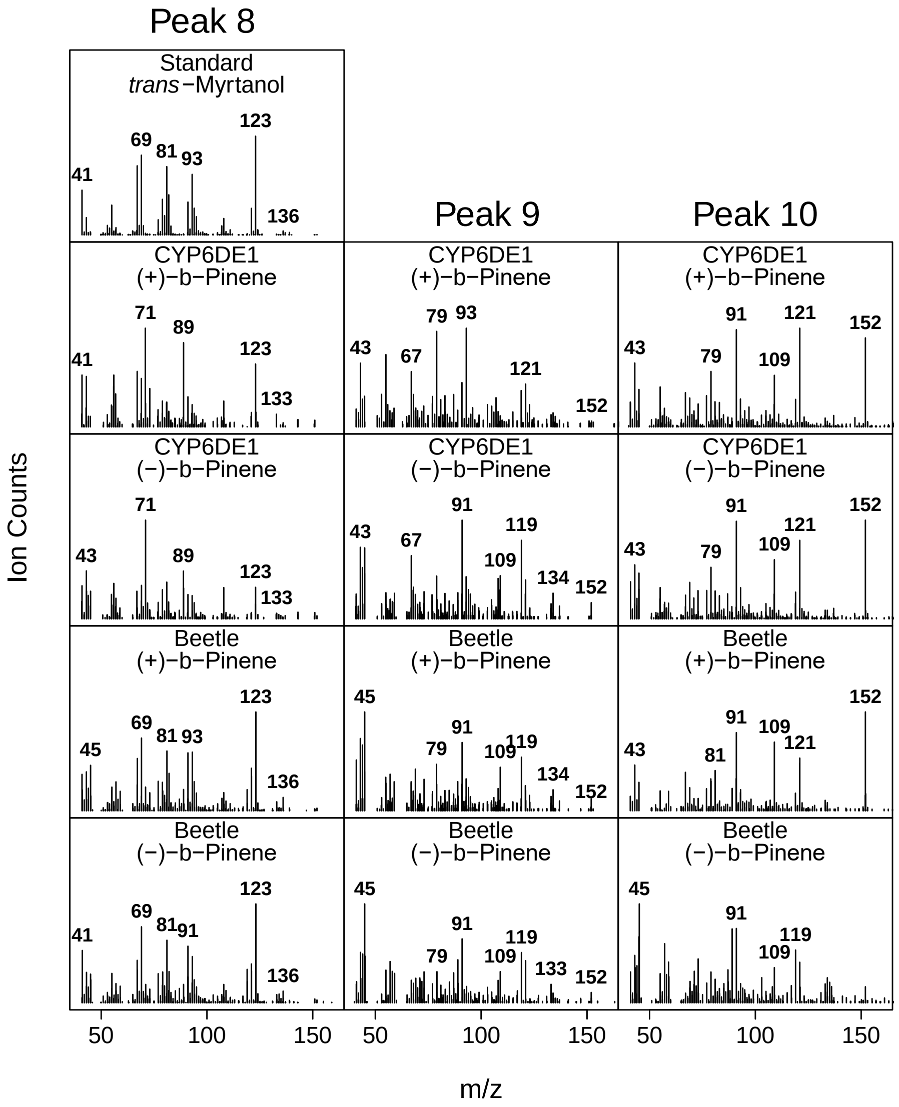

Figure S6 Mass spectra of peaks 8-10 from the gas chromatograms of extracts of CYP6DE1 or female beetles treated with (+) and (-)-β-pinene along with *trans*-myrtanol standards. Gas chromatograms with peak numbers can be found in Fig. 2B.

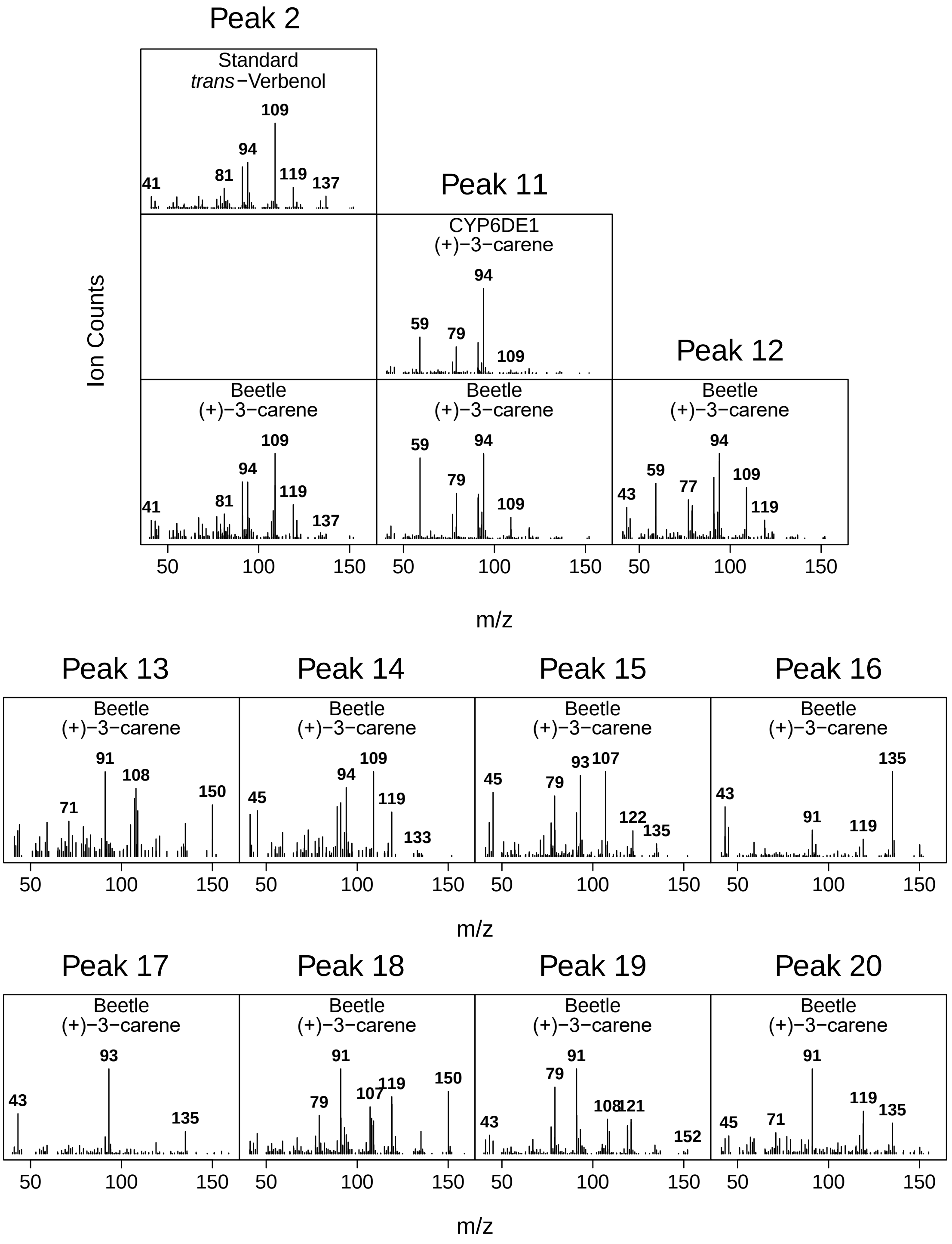

Figure S7 Mass spectra of peaks 2, 11-20 from the gas chromatograms of extracts of CYP6DE1 or female beetles treated with (+)-3-carene along with monoterpenol standards. Gas chromatograms with peak numbers can be found in Fig. 2C.

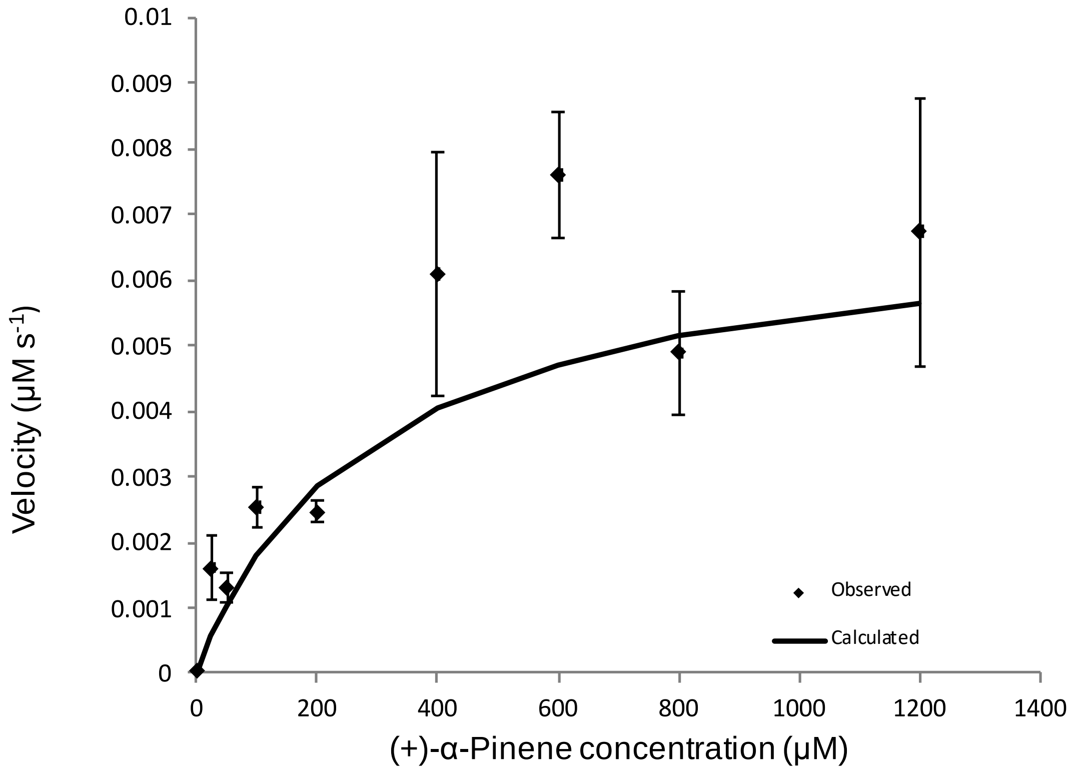

Fig S8 Michaelis-Menten saturation curve of CYP6DE1 with (+)-α-pinene as a substrate. Calculated Vmax, Km and kcat values are shown in Table 1.

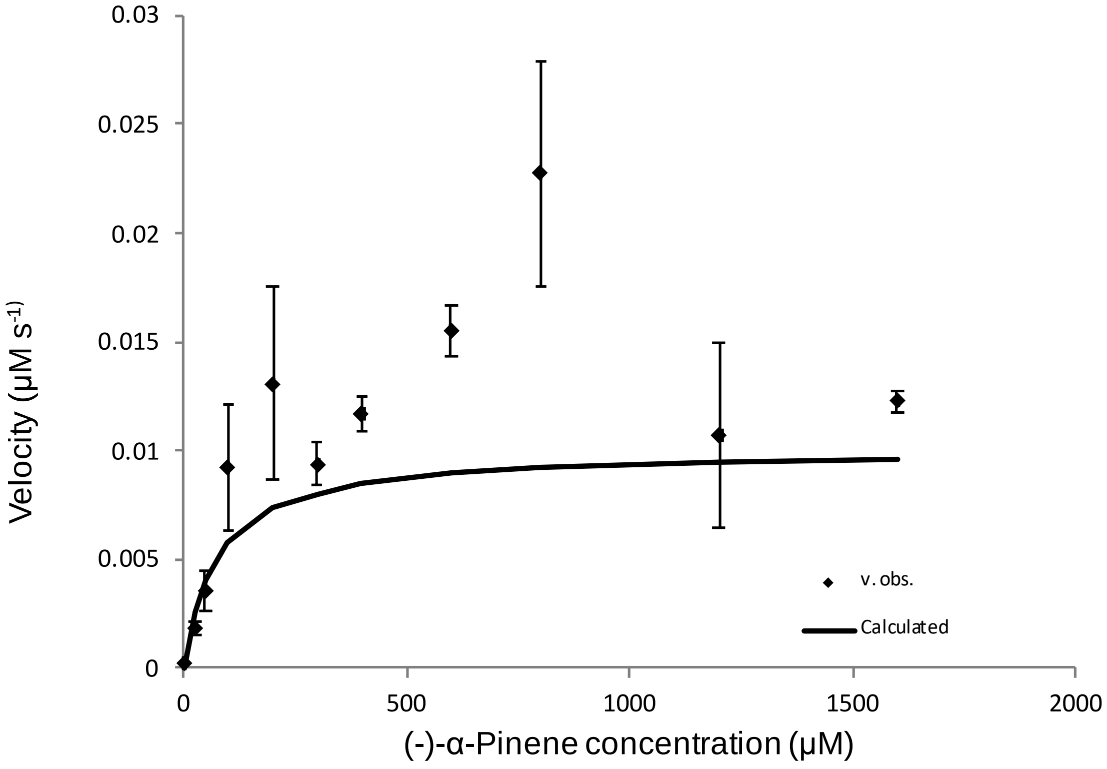

Fig S9 Michaelis-Menten saturation curve of CYP6DE1 with (-)-α-pinene as a substrate. Calculated Vmax, Km and kcat values are shown in Table 1.

Table S1. Substrates tested in *in vitro* assays for activity with CYP6DE1 and CYP6DE2.

Substrate	Activity Assay (*in vitro*)	
**Monoterpenes**	CYP6DE1	CYP6DE2
(+)-α-pinene	✓	✗
(-)-α-pinene	✓	✗
(+)-β-pinene	✓	✗
(-)-β-pinene	✓	✗
(+)-limonene	✗	✗
(-)-limonene	✗	✗
(+)-3-carene	✓	✗
β-phellandrene	✗	✗
myrcene	✗	✗
terpinolene	✗	✗
**Diterpene Resin Acids**		
abietic acid	✗	✗
neoabietic acid	✗	✗
dehydroabietic acid	✗	✗
palustric acid	✗	✗
isopimaric acid	✗	✗

Table S2. The retention index of all α-pinene, β-pinene and 3-carene products of CYP6DE1 and from extracts of MPB after treatment. All samples were injected onto a DB-Wax column. See Fig. 2 and S2-6 for the gas chromatograms and mass spectra of these peaks.

Reference	Retention Indices	Compound name
Peak 4	1553	β-pinene product
Peak 5	1569	β-pinene product
Peak 13	1637	3-carene product
Peak 8	1658	*trans* pinocarveol
Peak 1	1660	*cis* verbenol
Peak 2	1682	*trans* verbenol
Peak 11	1715	3-carene product
Peak 12	1723	3-carene product
Peak 14	1783	3-carene product
Peak 3	1799	myrtenol
Peak 15	1807	3-carene product
Peak 7	1817	β-pinene product
Peak 16	1835	3-carene product
Peak 6	1873	*trans* myrtanol
Peak 17	1880	3-carene product
Peak 18	1943	3-carene product
Peak 19	1955	3-carene product
Peak 9	2007	3-carene product
Peak 20	2075	β-pinene product
Peak 10	2093	β-pinene product
